# A Comparison of Relative Time to Peak and Tmax for Mismatch-Based Patient Selection

**DOI:** 10.3389/fneur.2017.00539

**Published:** 2017-10-13

**Authors:** Anke Wouters, Søren Christensen, Matus Straka, Michael Mlynash, John Liggins, Roland Bammer, Vincent Thijs, Robin Lemmens, Gregory W. Albers, Maarten G. Lansberg

**Affiliations:** ^1^Department of Neurosciences, Experimental Neurology, Leuven Research Institute for Neuroscience and Disease (LIND), KU Leuven, Leuven, Belgium; ^2^Laboratory of Neurobiology, Center for Brain and Disease Research, VIB, Leuven, Belgium; ^3^Department of Neurology, University Hospitals Leuven, Leuven, Belgium; ^4^Stanford Stroke Center, Stanford University Medical Center, Palo Alto, CA, United States; ^5^Florey Institute of Neuroscience and Mental Health, Heidelberg, VIC, Australia

**Keywords:** ischemic stroke, magnetic resonance imaging, perfusion imaging, thrombectomy, treatment

## Abstract

**Background and purpose:**

The perfusion-weighted imaging (PWI)/diffusion-weighted imaging (DWI) mismatch profile is used to select patients for endovascular treatment. A PWI map of Tmax is commonly used to identify tissue with critical hypoperfusion. A time to peak (TTP) map reflects similar hemodynamic properties with the added benefit that it does not require arterial input function (AIF) selection and deconvolution. We aimed to determine if TTP could substitute Tmax for mismatch categorization.

**Methods:**

Imaging data of the DEFUSE 2 trial were reprocessed to generate relative TTP (rTTP) maps. We identified the rTTP threshold that yielded lesion volumes comparable to Tmax > 6 s and assessed the effect of reperfusion according to mismatch status, determined based on Tmax and rTTP volumes.

**Results:**

Among 102 included cases, the Tmax > 6 s lesion volumes corresponded most closely with rTTP > 4.5 s lesion volumes: median absolute difference 6.9 mL (IQR: 2.3–13.0). There was 94% agreement in mismatch classification between Tmax and rTTP-based criteria. When mismatch was assessed by Tmax criteria, the odds ratio (OR) for favorable clinical response associated with reperfusion was 7.4 (95% CI 2.3–24.1) in patients with mismatch vs. 0.4 (95% CI 0.1–2.6) in patients without mismatch. When mismatch was assessed with rTTP criteria, these ORs were 7.2 (95% CI 2.3–22.2) and 0.3 (95% CI 0.1–2.2), respectively.

**Conclusion:**

rTTP yields lesion volumes that are comparable to Tmax and reliably identifies the PWI/DWI mismatch profile. Since rTTP is void of the problems associated with AIF selection, it is a suitable substitute for Tmax that could improve the robustness and reproducibility of mismatch classification in acute stroke.

## Introduction

The combination of MRI diffusion-weighted imaging (DWI) and perfusion-weighted imaging (PWI) maps is used to assess PWI/DWI mismatch, which provides an estimate of the volume of penumbral tissue and has shown promise in identifying patients with a favorable response to reperfusion (1, 2). There is, however, variability between studies in the assessment of the PWI/DWI mismatch. One area of variability is the type of PWI map used to identify critically hypoperfused tissue. The Tmax (time to the maximum of the residue function) map has gained popularity in recent endovascular stroke trials. Prior studies have shown that a Tmax delay of >6 s is a good predictor of critically hypoperfused tissue that is destined to infarction in the absence of timely reperfusion (2–6). The Tmax perfusion parameter primarily reflects the bolus delay between the site of the arterial input function (AIF) and the tissue (7). This delay sensitivity seems important, as Tmax has outperformed delay-corrected perfusion parameters such as cerebral blood flow (CBF) and mean transit time for identifying critically hypoperfused tissue (8–10). A drawback of Tmax is that calculation of this perfusion metric requires selection of an AIF (for deconvolution) and that the nature of the deconvolution algorithm renders the Tmax perfusion maps very sensitive to even minor changes in the shape of the AIF. Within patient variability in the AIF is unavoidable because the AIF is obtained by measuring the MRI signal in a few voxels in a main feeding artery (e.g., the middle cerebral artery) on the source perfusion images; a subjective process that results in profound variability in the shape of the AIF depending on which voxels are chosen. This in turn, causes variability in the Tmax perfusion maps and the Tmax lesion volumes. It also makes the Tmax map prone to errors resulting from imaging artifacts that perturb the AIF (11).

Time to peak (TTP) is a perfusion parameter that theoretically could be superior to Tmax for assessment of critically hypoperfused tissue because it does not require deconvolution and, therefore, is not dependent on an AIF. A potential drawback of TTP is that it is not only delay-sensitive (like Tmax) but also sensitive to arterial dispersion and tissue transit time. As a result, TTP reflects a sum of these three effects (12, 13). However, recently, it has been shown that summary parameters such as TTP display much less variability than AIF-based maps when properly normalized (11). Moreover, previous studies point to TTP in the range of 3–5 s as a sensitive and specific parameter to estimate penumbral tissue (8, 9, 14, 15). The most recent one, a combined MRI and PET study, showed that Tmax and TTP were the best predictors of penumbral tissue on PET, defined as CBF < 20 mL/100 g/min (9).

While these recent studies suggest that TTP and Tmax are both predictive of infarction, the impact on patient selection in clinical trials has not yet been compared (8, 9, 11). In this study, we used data from DEFUSE 2, a large prospective study, to compare relative TTP (rTTP) and Tmax in terms of image quality, lesion volumes, patient selection, and response to reperfusion among patients with a PWI/DWI mismatch (2).

## Materials and Methods

Imaging data were obtained from DEFUSE 2 (NCT01327989), a multicenter prospective cohort study. The local institutional review boards from all participating institutions approved the study. All subjects or legal representatives gave their informed consent. Study design and primary results are reported elsewhere (2). All patients eligible for the original study were included. Briefly, an MRI scan with PWI and DWI sequences was obtained on admission to classify patients according to target mismatch status. Definitions of target mismatch, malignant profile, reperfusion, and favorable clinical response at day 30 were adopted from the original DEFUSE 2 study (Table 1).

**Table 1:** Definitions used in the text.

Tmax	Time to the maximum of the residue function
Time to peak (TTP)	Time to the peak of the concentration time curve
Relative TTP (rTTP)	rTTP = TTP normalized by subtraction of the median TTP of the contralateral hemisphere
Target mismatch	A ratio between the volumes of critically hypoperfused tissue (Tmax > 6 s) and the ischemic core (Apparent Diffusion Coefficient < 620 × 10 mm^2^/s) of 1.8 or more, with an absolute difference of 15 mL or more; ischemic core volume of less than 70 mL; and less than 100 mL of tissue with a severe delay in bolus arrival (Tmax > 10 s)
Malignant profile	Ischemic core volume of more than 70 mL and/or more than 100 mL of tissue with a severe delay in bolus arrival (Tmax > 10 s)
Favorable clinical response	An improvement in the National Institute of Health Stroke Scale Score of eight points or more between baseline and day 30 or a score of 0–1 at day 30

We generated Tmax—and DWI—maps using a research version of the RAPID software (v2.5) with a customized Matlab plug-in for our rTTP calculation (Mathworks, Natick, MA, USA) (16). TTP maps were created by smoothing the tissue concentration time curve of each voxel by a 3-point running average filter followed by a spline interpolation to 0.5 s time resolution. The TTP was then recorded as the time of the peak of this smoothed tissue concentration time curve. Using a manually positioned midline plane in 3D space, we extracted the median TTP of tissue contralateral to the stroke (contralateral to the DWI lesion or, when no DWI lesion was present, the TTP deficit). The TTP map was then normalized by subtraction of the contralateral median TTP from the absolute TTP in each voxel, yielding a map (rTTP). All preprocessing steps, including masking, source image smoothing, motion correction, and segmentation, were identical for rTTP and Tmax.

We compared differences between rTTP and Tmax, including (1) the number of cases that were uninterpretable due to excessive artifacts; (2) the number of cases that required any artifact removal; (3) the mean volume of artifact removal in these cases; (4) correlation and absolute difference between rTTP and Tmax lesion volumes; (5) agreement in target mismatch assessment; and (6) the response to reperfusion for patients with and without target mismatch. For this final analysis, we only included patients in whom rTTP, Tmax, and reperfusion status could be assessed.

To compare rTTP and Tmax, we first determined the rTTP thresholds that yielded lesion volumes, which corresponded most closely with the lesion volumes obtained with the Tmax thresholds used in the DEFUSE 2 trial (>6 s for critically hypoperfused tissue and >10 s for severely hypoperfused tissue). These rTTP thresholds were defined as the values at which the median difference between rTPP and Tmax lesion volumes was closest to 0. This optimization analysis was performed after imaging artifacts had been manually removed. All subsequent analyses were based on these optimal rTTP thresholds.

### Statistical Analyses

Chi square test was used to compare categorical variables and Mann–Whitney *U* for continuous variables. Paired volumetric data was compared with the non-parametric Wilcoxon Signed Rank test. Cohen’s Kappa (κ) was calculated to express the degree of agreement between rTTP and Tmax for target mismatch classification. The association between reperfusion and favorable clinical response in patients with or without the target mismatch profile was compared with a multivariate logistic regression analysis with favorable clinical response on day 30 as the dependent variable. Explanatory variables were the DWI-volume (log transformed), age, target mismatch, reperfusion status, and an interaction term between target mismatch and reperfusion. Results were considered statistically significant at a *p*-value < 0.05. Analyses were done using R software (R Development Core Team (2008). R: A language and environment for statistical computing. R Foundation for Statistical Computing, Vienna, Austria).

## Results

In this study, we reanalyzed MRI scans of 110 patients from DEFUSE 2 who received endovascular therapy. Excessive imaging artifacts rendered both Tmax and rTTP maps uninterpretable in six patients (5%). The reasons for these artifacts were severe patient motion in four and a failed contrast bolus injection in two patients. In an additional two patients, the Tmax map was uninterpretable due to poor AIF selection, while the rTTP map was of good quality.

**Figure 1:**
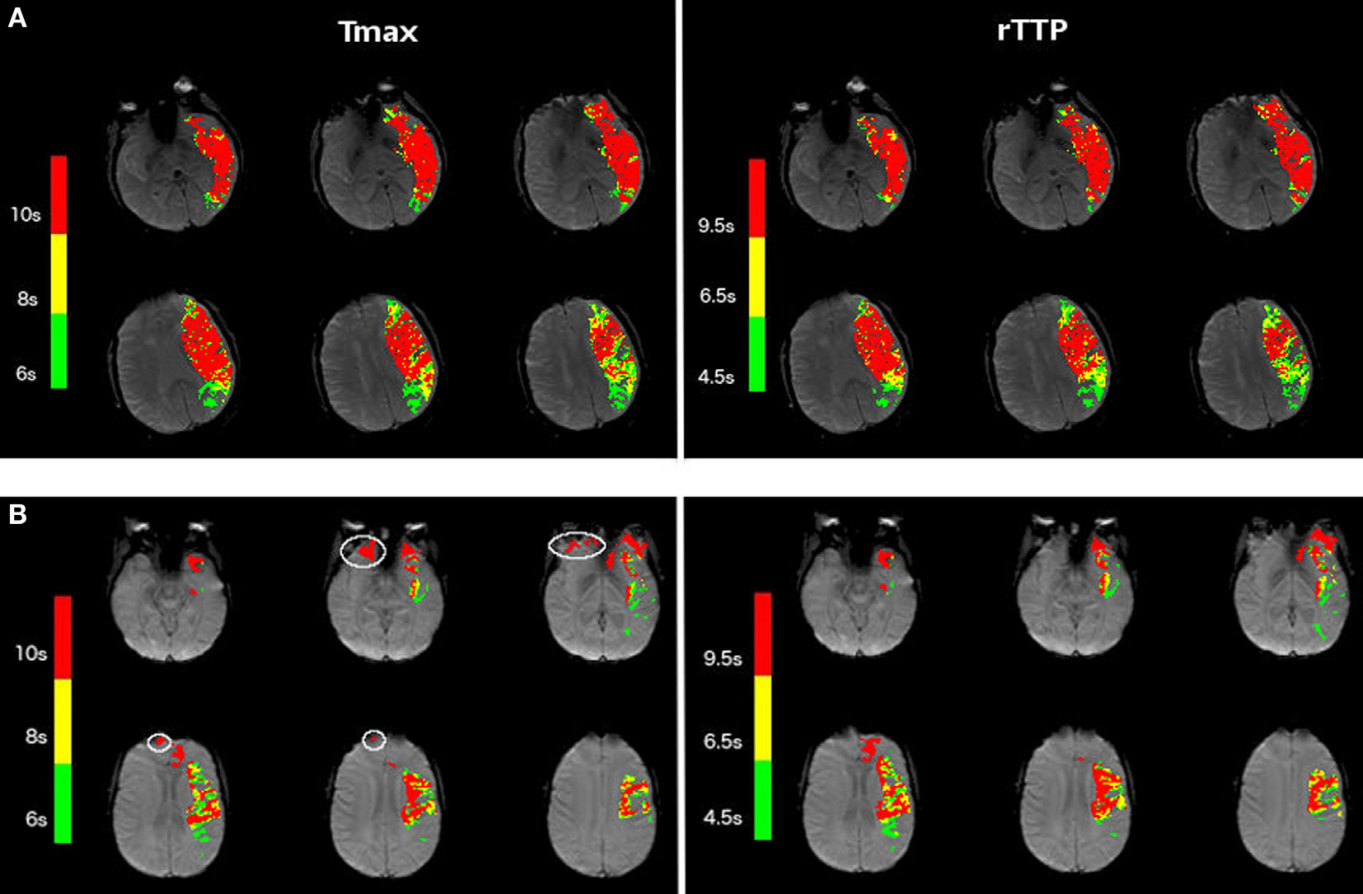
Examples of Tmax and relative TTP (rTTP) maps in acute stroke patients. **(A)** Shows the Tmax (left) and rTTP (right) perfusion maps of a patient with a left-hemispheric stroke, which illustrates the correspondence in volume and shape of the stroke lesion between Tmax and rTTP. **(B)** Illustrates another example of Tmax (left) and rTTP (right) perfusion maps in a patient with a left-sided stroke. White contours on the Tmax map depict small artifacts. This example illustrates that, in certain cases, the rTTP map is less susceptible to image artifacts.

Among the 102 patients with interpretable Tmax and rTTP perfusion maps, minor artifacts were manually removed from the Tmax maps in 24 patients (24%) and from the rTTP maps in 18 patients (18%; *p* for difference = 0.3) (Figure 1). Thirteen of these patients had artifacts on both Tmax and rTTP maps, 11 patients on Tmax alone, and five patients on rTTP alone. The mean clean-up volume was 14.2 mL for rTTP and 17.2 mL for Tmax (*p* = 0.7).

**Figure 2:**
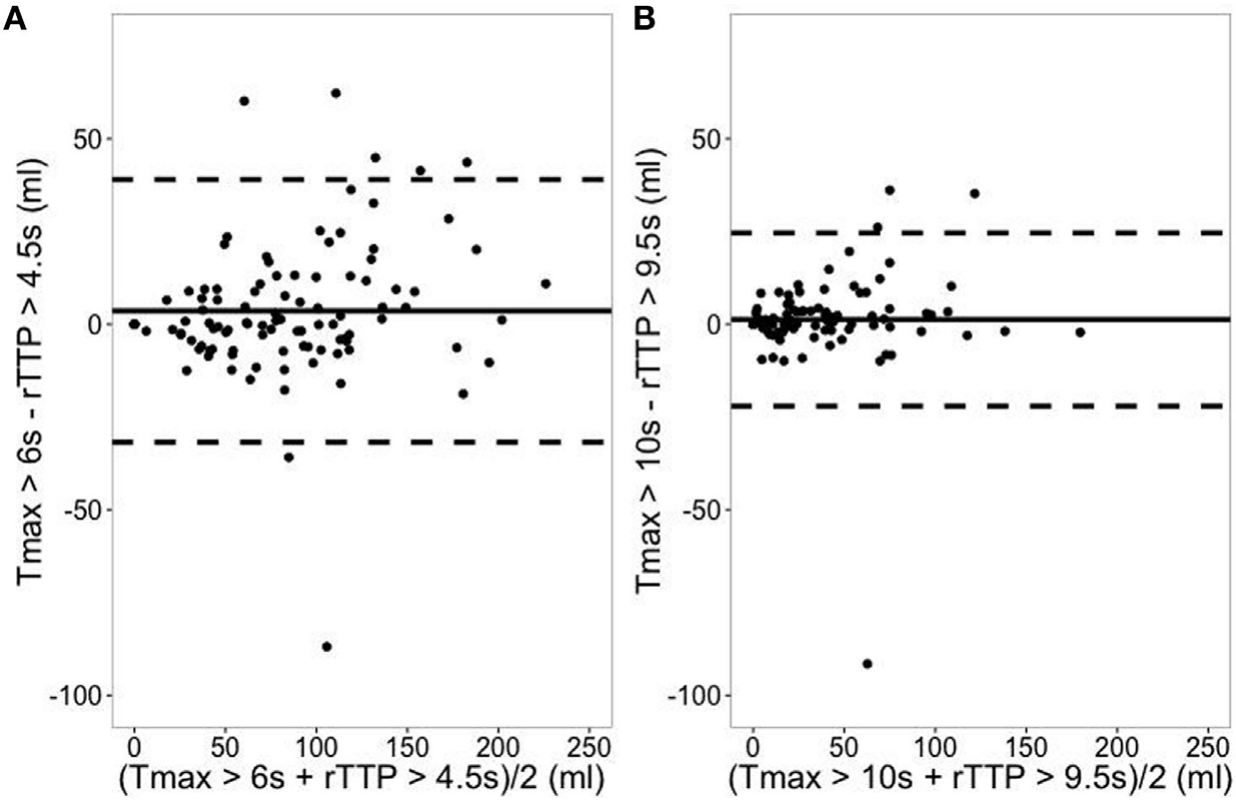
Correspondence between relative TTP (rTTP) and Tmax lesion volumes. **(A)** Represents the Bland–Altman plot for critical hypoperfusion. Solid line depicts the mean difference between Tmax > 6 s and rTTP > 4.5 s (mL) with its 95% prediction interval in dashed lines. **(B)** Represents the Bland–Altman plot for severe hypoperfusion. Solid line depicts the mean difference between Tmax > 10 s and rTTP > 9.5 s (mL) with its 95% prediction interval in dashed lines.

**Figure 3:**
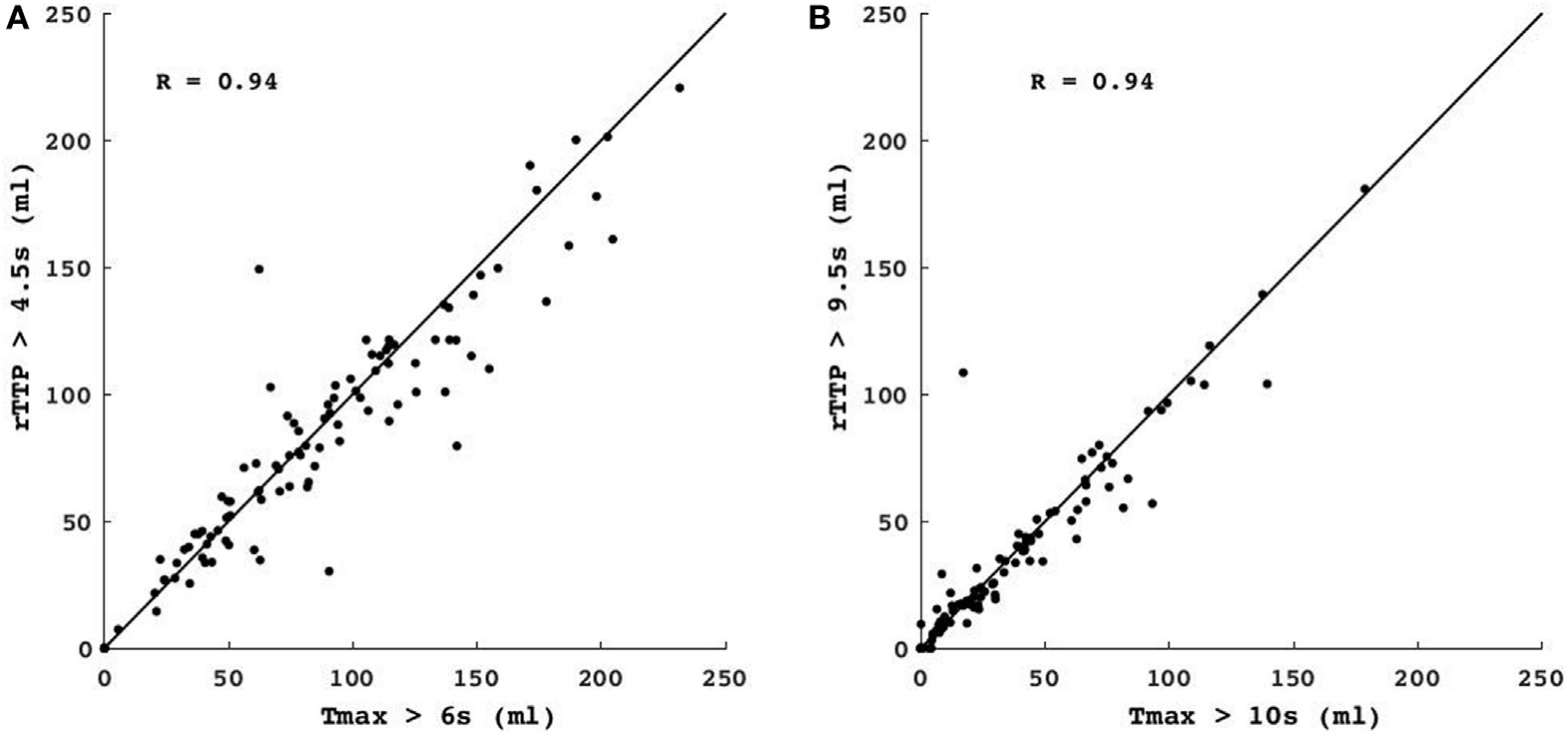
Comparison of Tmax vs. relative TTP (rTTP) lesion volumes. The scatterplots show the correlation between the volume of critical hypoperfusion assessed with a Tmax > 6 s vs. an rTTP > 4.5 s threshold **(A)** and the volume of severe hypoperfusion assessed with a Tmax > 10 s threshold vs. an rTTP > 9.5 s threshold **(B)**. Identity lines are depicted for both graphs. R = Pearson’s correlation coefficient.

Following artifact removal, Tmax > 6 s corresponded best with rTTP > 4.5 s (median difference between Tmax and rTTP lesion volumes 0.1 mL, *p* = ns) and Tmax > 10 s corresponded best with rTTP > 9.5 s (median difference −0.3 mL, *p* = ns) (Table 2; Figure 2). Pearson correlation coefficients (R) between Tmax and rTTP volumes were 0.94 for both critical and severe hypoperfusion (Figure 3). An example of the close correspondence between the rTTP and Tmax maps is presented in Figure 1.

**Table 2:** Comparison of rTTP and Tmax lesion volumes for different rTTP thresholds.

	Difference between rTTP and Tmax > 6 s, mL	Absolute difference between rTTP and Tmax > 6 s, mL	Relative difference between rTTP and Tmax > 6 s, %
rTTP > 4 s	14.1 (5.2, 22.0)	15.9 (7.5, 23.2)	20.7 (10.4, 33.7)
rTTP > 4.5 s	−0.1 (−9.5, 5.5)	6.9 (2.3, 13.0)	11.1 (3.8, 20.2)
rTTP > 5 s	−7.2 (−17.3, 0)	8.2 (4.0, 19.3)	13.0 (6.3, 24.8)

	**Difference between rTTP and Tmax > 10 s, mL**	**Absolute difference between rTTP and Tmax > 10 s, mL**	**Relative difference between rTTP and Tmax > 10 s, %**

rTTP > 9 s	1.1 (−0.4, 4.1)	3.0 (0.8, 6.5)	3.8 (1.3, 7.9)
rTTP > 9.5 s	−0.3 (−3.5, 1.1)	2.3 (0.6, 5.9)	3.2 (1.3, 7.5)
rTTP > 10 s	−2.1 (−6.3, 0)	3.8 (0.9, 7.4)	4.9 (1.8, 9.7)

*All represented data are median (IQR). Differences are calculated as rTTP − Tmax, absolute differences as |rTTP−Tmax|, and relative difference as |rTTP−Tmax|/Tmax*.

*IQR, interquartile range; rTTP, relative time to peak; Tmax, time to the maximum of the residue function*.

In 96 of the 102 patients (94%, κ = 0.82), there was agreement between target mismatch profiles assessed with Tmax and rTTP maps: 79 of these patients had the target mismatch profile and 17 did not. In six patients, the target mismatch profile classification differed between Tmax and rTTP. Two patients changed from target mismatch on Tmax to “no target mismatch” on rTTP and another four patients changed from “no target mismatch” on Tmax to target mismatch on rTTP. All six classification changes were the result of small differences in the lesion volumes between Tmax and rTTP (median difference 9.5 mL, IQR −8.05–13.8; see Table 3 for details).

**Table 3:** Overview of patients whose PWI/DWI mismatch classification differs depending on the use of Tmax vs. rTTP.

Patients	DWI lesion volume (mL)	rTTP > 4.5 s—Tmax > 6 s (mL)	Tmax/DWI target mismatch	rTTP/DWI target mismatch	rTTP/DWI mismatch criteria that changed target mismatch classification
1	23.6	6.7	No	Yes	Relative mismatch > 1.8
2	48.9	12.3	No	Yes	Relative mismatch > 1.8
3	48.5	17.7	No	Yes	Relative mismatch > 1.8
4	8.4	12.5	No	Yes	Absolute mismatch > 15 mL
5	52.7	−12.7	Yes	No	Relative mismatch < 1.8
6	2.3	−6.5	Yes	No	Absolute mismatch < 15 mL

*Tmax, time to the maximum of the residue function; rTTP, relative Time to Peak; DWI, diffusion-weighted imaging; PWI, perfusion-weighted imaging*.

We analyzed the effect of reperfusion on the 30 day favorable clinical response rate (the primary outcome for the DEFUSE 2 trial) in patients with and without target mismatch. When target mismatch status was assessed with Tmax, the odds ratio (OR) for favorable clinical response after reperfusion was 7.4 (95% CI 2.3–24.1) for patients with target mismatch and 0.4 (95% CI 0.1–2.6) for patients without. When target mismatch status was assessed with rTTP, these ORs were 7.2 (95% CI 2.3–22.2) and 0.3 (95% CI 0.1–2.2), respectively. The difference in ORs between patients with and without TMM was significant for both the Tmax and the rTTP-based analysis (*p* < 0.01).

## Discussion

In this study, rTTP thresholds of 4.5 and 9.5 s corresponded to the Tmax definitions for critical (Tmax > 6 s) and severe (Tmax > 10 s) hypoperfusion used in prior studies. The rTTP > 4.5 s threshold for critical hypoperfusion falls within the range of thresholds previously determined based on PET imaging (3–5 s), and is in very good concordance with the >4.8 s rTTP threshold determined in a recent study that used PET as the gold standard (9, 14, 15). The rTTP threshold of >9.5 s for severe hypoperfusion is novel since no previous studies have investigated rTTP thresholds that are comparable to Tmax > 10 s. Reanalysis of the primary DEFUSE 2 study results using these rTTP thresholds to identify patients with target mismatch yielded similar results as when Tmax thresholds were used.

In two patients, the Tmax maps were uninterpretable whereas the rTTP maps were of sufficient quality to determine target mismatch status. In these patients, the selection of the AIF failed, underscoring the advantages of rTTP since no AIF selection is required. Although AIF selection can be improved with engineering solutions, AIF selection, whether by humans or software, will remain a subjective choice that renders Tmax maps sensitive to small modifications in how this choice is made. Previous studies have shown that the location where the AIF is measured can highly influence Tmax perfusion volumes (17–19). Further, partial volume effects can lead to erroneous AIF measurements (20). Since calculation of rTTP does not require knowledge of the AIF, automated generation of rTTP maps may enable mismatch profiling when automated Tmax processing fails. Our study demonstrates in a large prospective cohort of stroke patients, this advantage of rTTP is observed in a small percentage (2%) of cases.

Both Tmax and rTTP are generated with respect to reference signals. Tmax is generated with the AIF as reference and rTTP is generated with the median TTP value in the contralesional hemisphere as reference. The rTTP reference is robust because it is the median of many observations. To illustrate this, consider a scan–rescan scenario with no change to the patient’s hemodynamics or the injection. In this case, the reference TTP value would be virtually identical between scans given the high number of voxels in which noise will average out. In contrast, the AIF is not robust because it is derived from the signal intensity in just a few (4–5) voxels. In a scan–rescan scenario, even minor changes in head position would result in selection of different voxels for the AIF and standard image noise would result in different signal intensities even if identical voxels were selected (11). Consequently, the reference AIF will vary between scans and hence the Tmax maps and Tmax lesion volumes will vary as well, whereas the rTTP maps will not. An additional advantage of rTTP over Tmax is that the rTTP calculation is more straightforward and, therefore, easier for vendors to implement in an identical fashion across software solutions. Consequently, variability between software solutions in Tmax maps (and thus lesion volumes) will be greater than variability in rTTP maps.

The simplicity of the rTTP calculation comes at a potential cost. rTTP is a parameter, which is influenced by several aspects of the bolus passage, including arterial dispersion, tracer arrival delay, and tissue transit time. In contrast, Tmax is primarily sensitive to tracer arrival delays. It might be counterintuitive that a summary parameter such as rTTP is at least of similar quality compared to Tmax (8). However, studies have shown that inclusion of dispersion may be an advantage when identifying tissue at risk of infarction (8, 10, 11). Thus, the sensitivity of rTTP to multiple aspects of the bolus passage may in fact be a strength, making this parameter well-suited for estimating critically hypoperfused tissue.

Conditions that deserve special mention as they might influence perfusion measures include carotid stenosis and leukoariosis. In the presence of a chronic carotid stenosis, delay-sensitive parameters (such as TTP and Tmax) will overestimate the amount of critically hypoperfused tissue. However, since rTTP and Tmax (calculated using a global AIF) are both delay-sensitive, the effect of carotid stenosis on these perfusion parameters is likely similar. While we lack information about carotid status in our study, prior studies have shown that the prolongation of bolus delay due to carotid stenosis is not clinically significant. One study in patients with acute MCA occlusions showed a median increase of only 1 s of Tmax delay between patients with and without ipsilateral carotid stenosis (21). Next, while it is well recognized that, in areas of leukoariosis, CBF is reduced (22, 23), there is likely little to no effect of leukoariosis on delay-sensitive parameters like TTP and Tmax.

It should be noted that although TTP maps have historically often been used to assess mismatch using a qualitative approach, the present analysis is a quantitative thresholding approach. We caution against patient selection using a qualitative review of the perfusion map, as this approach is prone to interrater variability and overestimation of tissue at risk.

Our study is limited to MR perfusion while many centers use CT perfusion to estimate core and penumbra in acute stroke patients. Future studies comparing Tmax to TTP would be required to support the use of TTP for assessment of critically hypoperfused tissue on CTP. Further, TTP is not a suitable parameter for estimating the ischemic core on CTP. The current standard for estimation of the ischemic core on CTP is CBF, and we do not expect that TTP can outperform CBF. Many software packages use an AIF for CBF calculation. Therefore, mismatch assessment on CTP would remain AIF dependent even if one were to substitute Tmax with TTP. It remains an open question whether non-AIF-dependent CBF techniques, such as the “maximal slope” method used by Siemens, can be as accurate as deconvolution-based CBF for estimating the core.

A limitation of the way we processed rTTP maps is that it required a manual step (to position the midline plane). Since this was a pilot study, no further automatization was pursued, but implementation of a fully automated analysis is easily feasible. Another limitation of this study is the lack of a gold standard to define the optimal rTTP threshold for critical hypoperfusion (tissue destined to go on to infarction in the absence of reperfusion). In this study, we used established Tmax thresholds to “calibrate” rTTP. Future research could use infarcts outlined on late follow-up MRI scans from patients without reperfusion as the gold standard for critical hypoperfusion. This was not possible in DEFUSE 2 due to the limited number of non-reperfused patients. It should also be noted that the use of late follow-up scans has its own limitations as there is no perfect time or method to accurately define the final infarct volume. For example, the presence of edema will lead to an overestimation of the infarct when the scan is obtained too early, whereas atrophy will lead to an underestimation when the scan is obtained late (24–26). Moreover, second strokes that occur during the follow-up period in the territory adjacent to the primary stroke can also complicate the accurate assessment of the final infarct volume.

In summary, this study, using a large prospective dataset, demonstrates that rTTP and Tmax provide comparable results in terms of lesion volumes and mismatch classification. Lesion volumes determined with rTTP thresholds of 4.5 and 9.5 s correspond closely with volumes obtained with previously identified Tmax thresholds for critical and severe hypoperfusion. Since the rTTP parameter is not AIF dependent and, therefore, not subject to the variability associated with AIF selection, it could serve as a substitute for Tmax that may improve the robustness and reproducibility of mismatch classification in acute stroke.

## Ethics Statement

The local institutional review boards from all participating institutions approved the study. All subjects or legal representatives gave their informed consent. Study design and primary results are reported elsewhere.

## Author Contributions

AW and RL: study concept and design, analysis and interpretation of data, drafting the manuscript. SC, GA, and ML: study concept and design, analysis and interpretation of data, drafting the manuscript, acquisition of data. MS, MM, and RB: study concept and design, revising the manuscript, acquisition of data. JL and VT: study concept and design, analysis and interpretation of data, revising the manuscript.

## Conflict of Interest Statement

AW receives a grant from European Union. SC performs consulting work for iSchemaView. RL is senior clinical investigator of FWO Flanders. GA has received consulting fees and expenses from Lundbeck for Steering Committee work and consulting fees from Concentric for serving on a Data Safely and Monitory Board. GA and RB are equity shareholders in iSchemaView and perform consulting work for iSchemaView. The other authors report no conflicts. The reviewer NY and handling editor declared their shared affiliation.
